# A Calcium Sensor Discovered in Bluetongue Virus Nonstructural Protein 2 Is Critical for Virus Replication

**DOI:** 10.1128/JVI.01099-20

**Published:** 2020-09-29

**Authors:** Shah Kamranur Rahman, Adeline Kerviel, Bjorn-Patrick Mohl, Yao He, Z. Hong Zhou, Polly Roy

**Affiliations:** a Department of Infection Biology, London School of Hygiene and Tropical Medicine, London, United Kingdom; b California NanoSystems Institute, UCLA, Los Angeles, California, USA; c Department of Microbiology, Immunology and Molecular Genetics, UCLA, Los Angeles, California, USA; Instituto de Biotecnologia/UNAM

**Keywords:** bluetongue virus, Ca^2+^, Ca^2+^ binding protein, casein kinase II subunit alpha, circular dichroism, CryoEM, nonstructural protein, reverse genetics, viral inclusion bodies

## Abstract

Many viruses use specific viral proteins to bind calcium ions (Ca^2+^) for stability or to modify host cell pathways; however, to date, no Ca^2+^ binding protein has been reported in bluetongue virus (BTV), the causative agent of bluetongue disease in livestock. Here, using a comprehensive bioinformatics screening, we identified a putative EF-hand-like Ca^2+^ binding motif in the carboxyl terminal region of BTV nonstructural phosphoprotein 2 (NS2). Subsequently, using a recombinant NS2, we demonstrated that NS2 binds Ca^2+^ efficiently and that Ca^2+^ binding was perturbed when the Asp and Glu residues in the motif were substituted by alanine. Using circular dichroism analysis, we found that Ca^2+^ binding by NS2 triggered a helix-to-coil secondary structure transition. Further, cryo-electron microscopy in the presence of Ca^2+^ revealed that NS2 forms helical oligomers which, when aligned with the N-terminal domain crystal structure, suggest an N-terminal domain that wraps around the C-terminal domain in the oligomer. Further, an *in vitro* kinase assay demonstrated that Ca^2+^ enhanced the phosphorylation of NS2 significantly. Importantly, mutations introduced at the Ca^2+^ binding site in the viral genome by reverse genetics failed to allow recovery of viable virus, and the NS2 phosphorylation level and assembly of viral inclusion bodies (VIBs) were reduced. Together, our data suggest that NS2 is a dedicated Ca^2+^ binding protein and that calcium sensing acts as a trigger for VIB assembly, which in turn facilitates virus replication and assembly.

**IMPORTANCE** After entering the host cells, viruses use cellular host factors to ensure a successful virus replication process. For replication in infected cells, members of the *Reoviridae* family form inclusion body-like structures known as viral inclusion bodies (VIB) or viral factories. Bluetongue virus (BTV) forms VIBs in infected cells through nonstructural protein 2 (NS2), a phosphoprotein. An important regulatory factor critical for VIB formation is phosphorylation of NS2. In our study, we discovered a characteristic calcium-binding EF-hand-like motif in NS2 and found that the calcium binding preferentially affects phosphorylation level of the NS2 and has a role in regulating VIB assembly.

## Abstract (precis)

After entering the host cells, viruses use cellular host factors to ensure a successful virus replication process. For replication in infected cells, members of the *Reoviridae* family form inclusion body-like structures known as viral inclusion bodies (VIB) or viral factories. Bluetongue virus (BTV) forms VIBs in infected cells through nonstructural protein 2 (NS2), a phosphoprotein. An important regulatory factor critical for VIB formation is phosphorylation of NS2. In our study, we discovered a characteristic calcium-binding EF-hand-like motif in NS2 and found that the calcium binding preferentially affects phosphorylation level of the NS2 and has a role in regulating VIB assembly.

## INTRODUCTION

Bluetongue virus (BTV) of the *Orbivirus* genus in the *Reoviridae* family is an insect-borne animal pathogen. BTV is vectored by *Culicoides* spp. and causes infection in vertebrate hosts (sheep, cattle, and goat) in many parts of the world that has considerable economic impact. The nonenveloped BTV particle is a complex icosahedral structure, consisting of seven structural proteins (VP1 to VP7) that are organized in an outer capsid and an inner capsid (core). The outer capsid is composed of two major proteins, VP2 and VP5, and is responsible for attachment and membrane penetration. Both proteins are lost during endocytosis, and the inner core is subsequently released into the cytoplasm. The BTV core consists of the remaining five proteins and the viral genome of 10 double-stranded RNA (dsRNA) segments. In addition to 7 structural proteins, 4 nonstructural proteins, NS1 to NS4, are synthesized during virus replication. Two of these are major nonstructural (NS) proteins, NS1 and NS2, which are synthesized during early infection, and each plays an essential role in virus replication. The third NS protein, NS3/NS3A, is a transmembrane protein and facilitates release of the newly assembled BTV (1–3). NS4 is newly identified, and its function is still not fully characterized and a fifth putative nonstructural protein (4–6). Previously, we reported that NS3 interacts with cellular exocytic pathway protein p11 (S100A10), a protein known to facilitate Ca^2+^ uptake, suggesting indirect involvement of NS3 with Ca^2+^-related signaling pathways (1, 7). Several enveloped and nonenveloped viruses employ their proteins (Tat, gp120, nef of HIV-1, HBx of hepatitis B virus [HBV], NSP4 of rotavirus, P7 of hepatitis C virus [HCV]) to modulate cellular Ca^2+^ hemostasis for ensuring a successful viral life cycle (8, 9). For example, rotavirus expresses membrane-localizing NSP4 protein that binds Ca^2+^ and influences Ca^2+^ homeostasis (9). BTV and rotavirus belong to the same family; however, unlike rotavirus NSP4, a Ca^2+^ binding protein in BTV is yet unknown.

In this study, we used bioinformatics to identify whether any of the BTV proteins has a Ca^2+^ binding motif. We identified EF-hand-like motif in NS2, which is the only viral encoded phosphoprotein and is essential for replication (10). The 357 amino acid (aa) long NS2 is the major component of viral inclusion bodies (VIBs), the sites for viral capsid assembly and genome packaging. The identified EF-hand-like motif in NS2 was comparable to those found in other member proteins of the EF-hand superfamily that are characteristically known for Ca^2+^ binding. Using recombinant purified protein, together with biochemical and biophysical analysis, we demonstrated that Ca^2+^ binding changes the secondary structural conformation of NS2. Moreover, our cryo-electron microscopy (CryoEM) analysis of NS2 oligomer in the presence of Ca^2+^ exhibited a helical architecture. By site-specific targeted mutagenesis in the recombinant NS2 and in the replicating viral genome by reverse genetics, we identified the specific Ca^2+^ binding site of NS2 and demonstrated its importance in NS2 phosphorylation level, formation of VIBs, and virus replication. Altogether, our results suggest that Ca^2+^ sensing by NS2 influences NS2 phosphorylation and may be involved in the regulation of VIB assembly/disassembly, a process critical for virus replication and the release of newly assembled cores from VIBs (2, 11).

## RESULTS

### Computer-based sequence analysis of BTV proteins reveals putative Ca^2+^ binding site in NS2.

**FIG 1:**
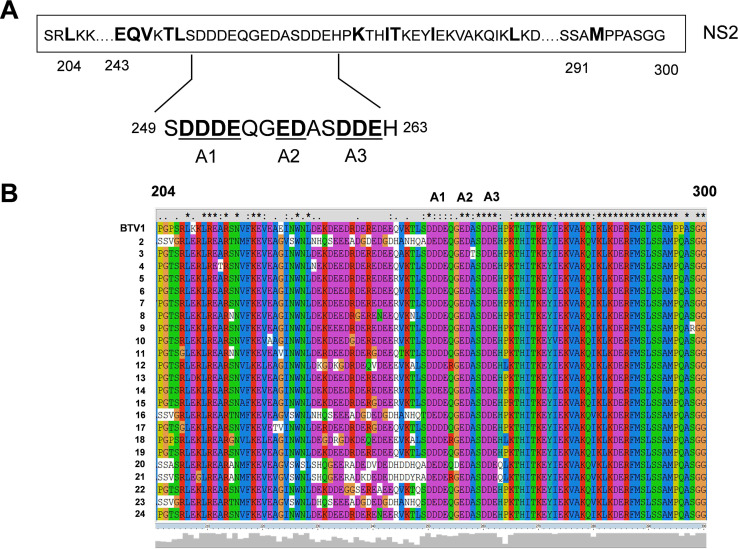
Sequence analysis of the BTV1 NS2 protein. (A) Amino acid sequence of NS2 (residues 204 to 300) with signature residues of the EF-hand motif highlighted in bold, and location of the three putative calcium binding sites (A1, A2, A3) containing aspartic acid and glutamic acid residues are indicated. (B) Amino acid sequence alignment showing the conservation of the three putative calcium binding sites in NS2 among 24 different BTV serotypes.

In order to identify putative Ca^2+^ binding motif, we used the SMART motif search program for each of the 11 BTV proteins, including seven structural proteins (VP1 to VP7) and four NS proteins (12). In our linear sequence search, only NS2 exhibited signature residues (aa 200 to 300) of EF-hand-like motif, found in Ca^2+^ binding proteins of the EF-hand superfamily (Fig. 1). In particular, presence of acidic amino acids Asp and Glu in the region aa 250 to 262 suggests the calcium binding potential of this segment of NS2 (Fig. 1A) (13–14). However, we found that the relative positioning of signature residues of the EF-hand motif and Ca^2+^ binding residues identified in NS2 is different than has been observed in a typical EF-hand containing calcium binding proteins (CaBP), thus making this putative motif less obvious. These acidic residues in NS2 are continuous rather than alternate as found in the case of standard EF-hand motifs (Fig. 1A). These clusters (aa 250 to 262) of Asp (D) and Glu (E) of NS2 resemble more closely the “Ca^2+^ bowl” found in BK (big potassium) channels (15–21). Further, these Asp and Glu residues are highly conserved among different BTV serotypes, indicating that the putative Ca^2+^ binding motif is likely to be important for BTV replication (Fig. 1B).

### Demonstration of Ca^2+^ binding ability of NS2 oligomers and helix-to-coil transition in secondary structure.

**FIG 2:**
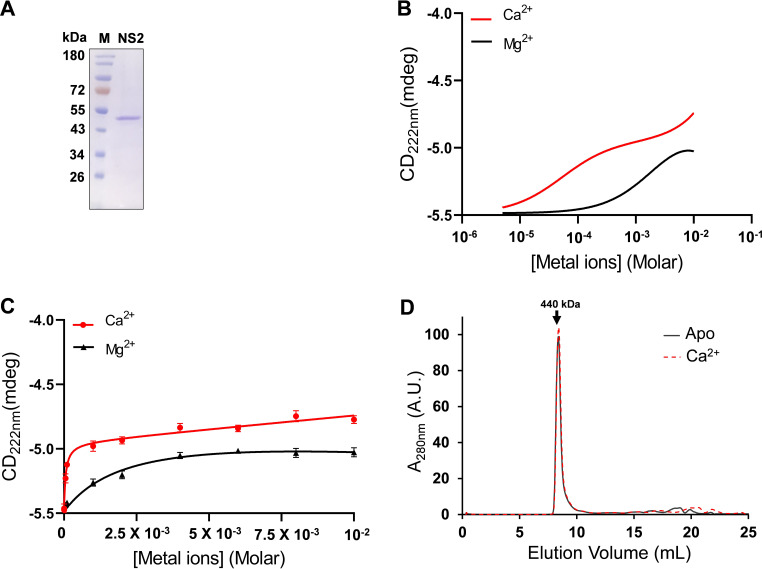
Calcium binding by NS2 and its oligomeric state. Purified NS2 was pretreated with Chelex 100 resin and then incubated with different Ca^2+^ concentrations (5 μM to 10 mM range) and analyzed by circular dichroism. (A) The NS2 protein was expressed in E. coli and analyzed by SDS-PAGE gel followed by Coomassie blue staining. M is the molecular mass markers as shown. (B) Far-UV CD spectra of Ca^2+^ titration binding by NS2 protein represented in log_10_ scale. Binding constant (*K_d_*) values of NS2 protein with Ca^2+^ (*K_d_* = 53.9 ± 8.4 μM; red) and Mg^2+^ (*K_d_* = 2.48 ± 0.4 mM; black). (C) The plot of Ca^2+^ titration binding in linear scale to show saturation points. (D) Size exclusion chromatography showing both apo NS2 (gray line) and in the presence of Ca^2+^ (red line) were eluted at the elution volume corresponding to a marker protein of 440 kDa.

To validate our bioinformatics analysis, we expressed NS2 in Escherichia coli in a nonphosphorylated form that was purified and analyzed by SDS-PAGE gel (Fig. 2A). Purified NS2, free of any bound metals and pretreated with Chelex 100, was examined for Ca^2+^ binding activity during metal titration experiment through circular dichroism (CD). The changes in intrinsic far-UV CD spectra were recorded, as the direct measure of Ca^2+^ binding to purified NS2, without (apo) or with (holo) Ca^2+^ at increasing concentrations of Ca^2+^ from 5 μM to 10 mM to calculate the dissociation constant, *K_d_*. NS2 showed a *K_d_* value of 53.9 (±8.4) μM for Ca^2+^ binding. In comparison, another divalent ion, Mg^2+^, when tested for NS2 binding in a parallel titration experiment with the same concentration range, showed a *K_d_* value of 2.48 (± 0.4) mM, suggesting a weaker binding of Mg^2+^ compared to Ca^2+^ (Fig. 2B and C).

**FIG 3:**
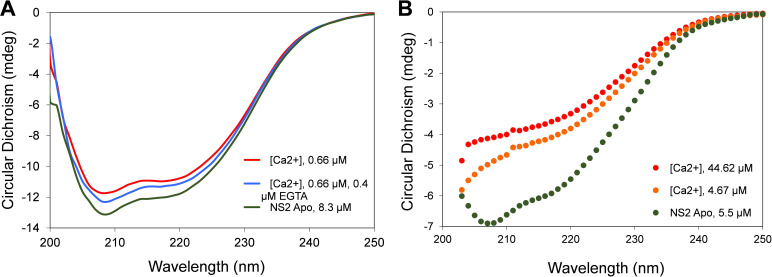
Helix-to-coil transition of NS2 in the presence of Ca^2+^. Analysis of CD spectra in the presence of Ca^2+^. (A) Far-UV spectrum of 8.3 μM NS2 apo alone (green line), in the presence of 0.66 μM Ca^2+^ (red line), and in the presence of 0.66 μM Ca^2+^ and 0.4 μM EGTA (cyan line). (B) Far-UV spectrum of 5.5 μM NS2 apo alone (green), in the presence of 4.67 μM Ca^2+^ (orange), and in the presence of 44.67 μM Ca^2+^ (red).

Also, NS2 formed stably intact oligomers and eluted at an elution volume on the size exclusion column corresponding to an estimated molecular weight of ∼440 kDa, suggesting decamers (Fig. 2D). The stability of the oligomers was not dependent on Ca^2+^ binding, as both apo NS2 and NS2-Ca^2+^ eluted at the same elution volume. To investigate further the effect of Ca^2+^ on NS2 secondary structure, which was largely alpha helical, we analyzed the far-UV CD spectrum at optimum Ca^2+^ concentrations based on the prior titration experiments (Fig. 3A). NS2 showed dose-dependent changes in secondary structure elements in the presence of Ca^2+^ (Fig. 3B).

**FIG 4:**
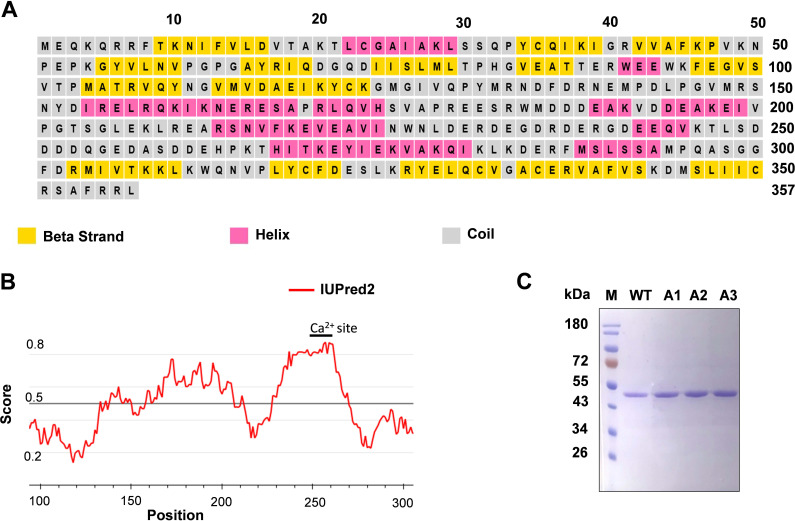
*S*econdary structure prediction of purified wild-type and Ca^2+^ mutant NS2 proteins. (A) Secondary structure prediction of NS2 using PSIPRED (23) showing amino acid regions for predicted beta strand (yellow), helix (pink), and coil (gray). (B) Intrinsic unfolded regions predicted by IUPred2 (24) shows high propensity of unfolding near and at the Ca^2+^ site (black line). The *x* axis shows amino acid positions, and the *y* axis shows probability score 0 to 1. (C) Coomassie blue-stained SDS-PAGE of the wt and NS2 mutant proteins. M is the molecular mass markers.

The effect of Ca^2+^ on NS2 spectra was diminished in the presence of 0.4 μM EGTA, consistent with chelation of the Ca^2+^ ion (Fig. 3A) (18). Importantly, at a higher molar concentration of Ca^2+^ ([Ca^2+^] = 44.67 μM), NS2 showed a very different CD spectrum (Fig. 3B) suggestive of a helix-to-coil transition in response to Ca^2+^ binding, as shown by other Ca^2+^ binding proteins from the EF-hand superfamily, for example, calmodulin (22). The change of helix to coil also prompted us to analyze *in silico* predicted secondary structure of NS2 near the Ca^2+^ binding site. The computer program PSIPRED suggested helix, beta strands, and coils in NS2 protein in different regions; however, IUPred2, a specific program to predict intrinsic unfolded regions or coil, suggested unfolded regions are mainly located near and at the Ca^2+^ binding site (Fig. 4A and B) (23, 24).

### Identification of Ca^2+^ binding site of NS2.

**FIG 5:**
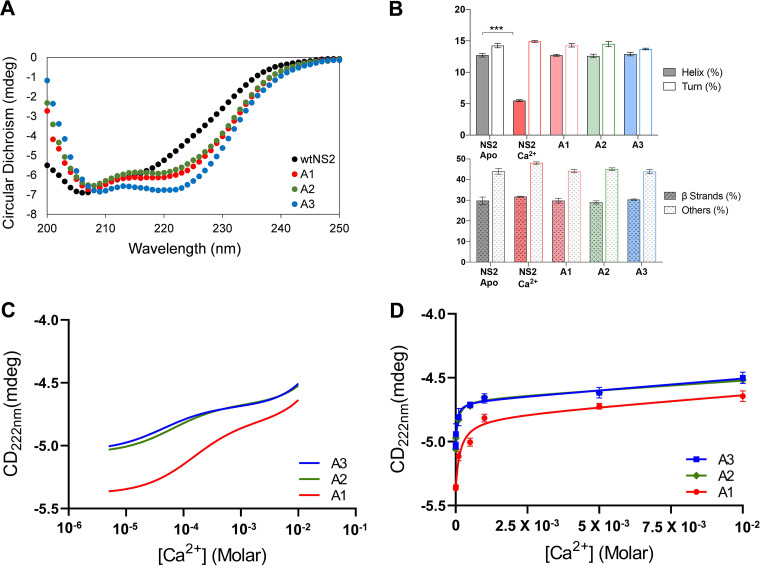
CD spectra and Ca^2+^ binding by NS2 mutants. Purified wtNS2 and mutants A1, A2, and A3 were analyzed by circular dichroism (A) Comparative far-UV CD spectra of wtNS2 (black circles) and mutants A1 (red), A2 (green), and A3 (cyan). (B) Estimation of percent secondary structure contents from far-UV CD spectra (*n* = 3). Other predicted secondary structure elements, such as 3-10 helices, bends, irregular/loops, and π-helices are represented as “others.” A star (*) denotes a significant difference from control (*P < *0.05) (*n* = 3). (C) Far-UV CD spectra of Ca^2+^ titration binding by NS2 mutant proteins represented in log_10_ scale. The *K_d_* (μM) values of mutants A3 (*K_d_* = 44 ± 2.4; cyan), A2 (*K_d_* = 64 ± 1.8; green), and A1 (*K_d_* = 150 ± 3.2; red). (D) The plot of Ca^2+^ titration in linear scale to show saturation points.

To determine which Asp and Glu residues in the predicted region of aa 250 to 262 are responsible for Ca^2+^ binding activity, we generated a series of recombinant NS2 mutant proteins by site-specific mutagenesis targeting these three sites (Fig. 1; Table 1). The amino acid substitutions were introduced by replacing negatively charged residues Asp (D) and Glu (E) with Ala (A), a neutral amino acid, which would have minimal impact on overall protein structure, unlike deletion mutants. The NS2 alanine mutant proteins, such as DDDE_250–253_AAAA (A1), ED_256–257_AA (A2), and DDE_260–262_AAA (A3), respectively, were then purified and analyzed by gel electrophoresis to determine that each mutant protein expressed was stable and equivalent to wild-type NS2 (wtNS2) (Fig. 4C). Further, size exclusion chromatography analysis of each protein showed that all three mutant proteins appeared equivalent to wtNS2, with an approximate molecular mass of ∼440 kDa (data not shown). Prior to investigation of Ca^2+^ binding activity of NS2 mutants, we compared their CD spectra with that of wtNS2 and calculated the estimated secondary structure elements using the BeStSel program (25) (Fig. 5A). There was no significant change in the percentage of helix, beta strands, or turns in mutants, indicating no major changes in the secondary structure elements (Fig. 5B). CD titration analysis of each NS2 mutant protein was then performed in the presence of Ca^2+^. The two mutants A2 and A3 showed the *K_d_* values (∼64 μM and ∼44 μM, respectively) similar to that of wtNS2 (*K_d_* value of ∼54 μM), suggesting not much change in the Ca^2+^ binding activities of mutants A2 and A3 (Fig. 5C and D). However, the mutant A1 showed weak Ca^2+^ binding as reflected from an increase in *K_d_* value to ∼150 μM (Fig. 5C and D). Thus, the four alanine residue (aa 250 to 253) substitutions of Glu and Asp residues have affected Ca^2+^ binding ability significantly, indicating that the three consecutive Glu and an Asp DDDE at aa 250 to 253 are important for Ca^2+^ binding activity of NS2.

**TABLE 1:** Alanine substitution mutations of NS2

Alanine mutant	Abbreviation
S_249_A + S_259_A	SAA
DDDE_250–253_AAAA	A1
ED_256–257_AA	A2
DDE_260–262_AAA	A3
DDDE_250–253_AAAA + ED_256–257_AA	A1 + A2
DDDE_250–253_AAAA + ED_256–257_AA + DDE_260–262_AAA	A 1 + A2 + A3

### Ca^2+^ binding enhances phosphorylation level of NS2 *in vitro*.

**FIG 6:**
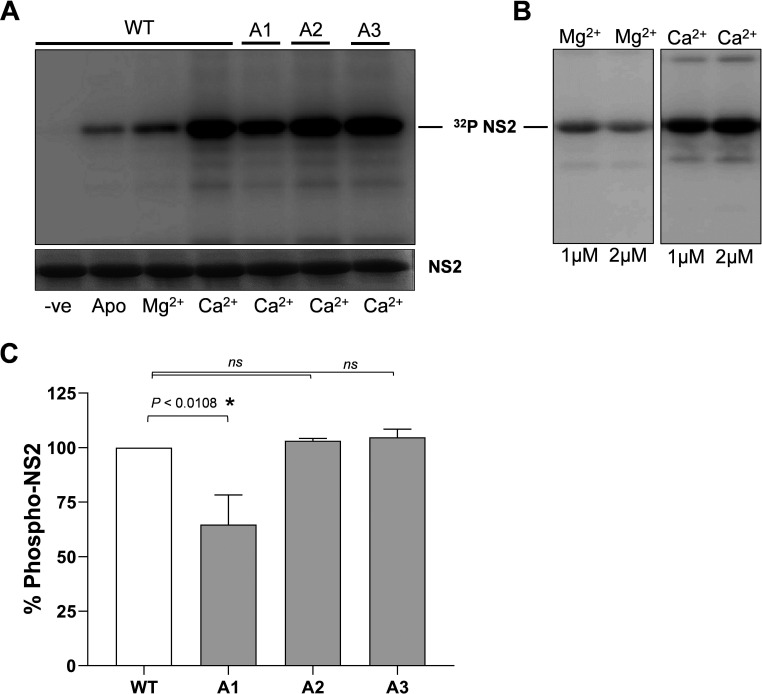
Phosphorylation of NS2 increases in the presence of calcium during *in vitro* kinase assay. Phosphorylation levels of NS2 by CK2 kinase was determined by the intensity of γ-^32^P signal transferred to NS2. (A) wtNS2 phosphorylation levels in the absence of CK2α (negative control, −ve) with CK2α before metal binding (Apo) and in the presence of Mg^2+^ and Ca^2+^. Phosphorylation levels in NS2 mutants A1, A2, and A3 in the presence of Ca^2+^. NS2 loading control is shown in the lower panel. (B) NS2 phosphorylation levels in the presence of different concentrations (1 μM or 2 μM) of Mg^2+^ (left) and Ca^2+^ (right). Both blots were scanned together. (C) Densitometry analysis of blot for wild-type NS2 and mutants in the presence of Ca^2+^ from panel A. The *P* value was determined from the *t* test of values for γ-^32^P intensity (*n* = 3). ns, Not significant.

Since the Ca^2+^ binding site (aa 250 to 253) is in juxtaposition of serine residues (S249, S259), known for phosphorylation modification, it was more likely that Ca^2+^-mediated transition in the secondary structure elements could affect the level of NS2 phosphorylation. Therefore, we undertook an *in vitro* kinase assay for NS2 phosphorylation, a modification required for VIB assembly (2, 11). Since casein kinase II alpha (CK2α) is responsible for NS2 phosphorylation, we used CK2α and purified unmodified NS2 as substrate (11). Our data showed that, for the fixed ratio of substrate and kinase, a much higher signal of [γ-^32^P]-labeled phosphate group transfer to NS2 was achieved in the presence of Ca^2+^ ions than that in the presence of Mg^2+^ ions (Fig. 6A and B). Presence of the Mg^2+^ ion showed a minimum basal level of phosphorylation of substrate NS2, determined at two different metal ion concentrations. Taken together, of these two ions, Ca^2+^ binding specifically increases phosphorylation level of purified NS2 protein. Further, we did not observe any increase in the activity of CK2α for another substrate (e.g., glutathione *S*-transferase [GST]) in the presence of Ca^2+^ ions (control; data not shown). To confirm further the specificity of Ca^2+^ binding activity on NS2 phosphorylation, we assessed the three NS2 mutants A1, A2, and A3 as substrates for CK2α kinase assay in the presence of Ca^2+^ (Fig. 6C). The NS2 mutants A2 and A3 did not show any significant change in the level of phosphorylation compared to that of wtNS2. In contrast, NS2 mutant A1 showed a marked decrease in the level of phosphorylation, suggesting a critical role of the calcium binding site on phosphorylation of the protein by the CK2α kinase (Fig. 6C).

### Subunits in NS2 oligomers are arranged in helical fashion.

**FIG 7:**
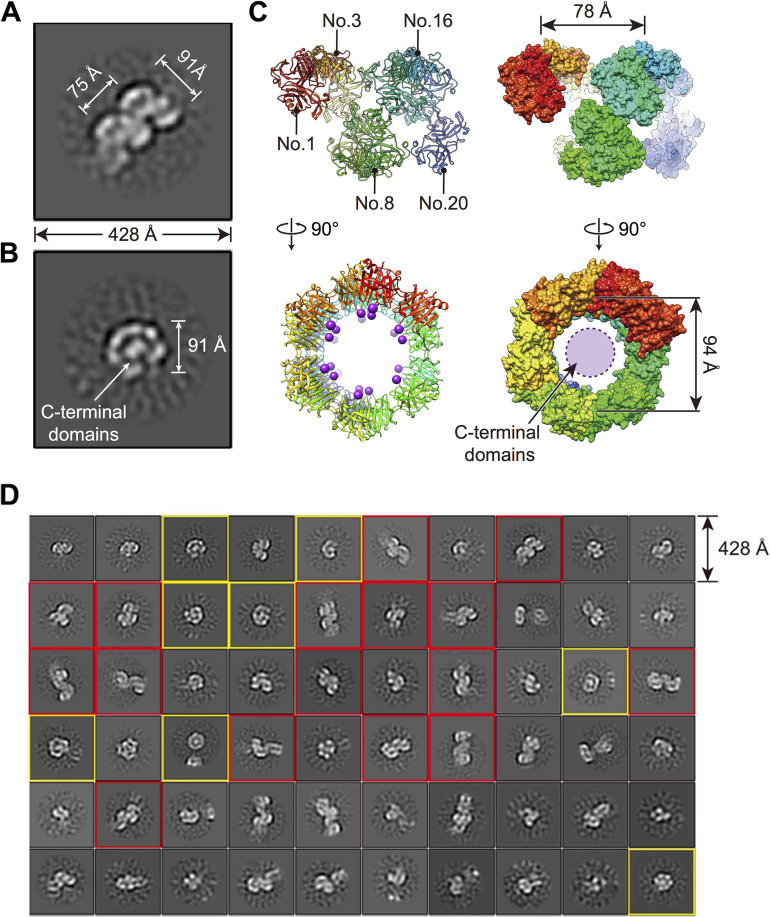
Cryo-electron microscopy of NS2 oligomer. (A and B) Representative class averages of CryoEM side (A) and front (B) views of NS2. The pitch and diameter of NS2 helix are measured based on the 2D class average results. (C) Ribbon and surface representations of oligomeric NS2 N-terminal domains. The model is generated based on the crystal structure of NS2 N-terminal domain (PDB accession number 1UTY) (22) and related crystal packing information. The C-terminal ends of each NS2 N-terminal domain (residue K160) are labeled as purple spheres. (D) CryoEM 2D classification result of 159,361 particles. 2D classes of NS2 particles on their side and front views are boxed in red and yellow, respectively. Numbers 1, 3, 8,16, and 20 denote subunits of N-terminal domain.

Since NS2-Ca^2+^ interaction changes the percentage of helix in NS2, we investigated whether this change has any destabilizing effect on the oligomeric state of NS2. To this end, purified NS2 in the presence of Ca^2+^ was visualized by cryo-electron microscopy (CryoEM). In total, 159,361 particle images were selected from 2,712 CryoEM micrographs and subjected to image classification to obtain two-dimensional (2D) class averages. Particles with clear helical organizational features were observed in some 2D classes (Fig. 7). The pitch and outer diameter of those helical particles were measured to be 75 Å and 91 Å, respectively (Fig. 7A and B), matching the parameters of the helical structure of NS2 N-terminal domain observed by X-ray crystallography (26). Modeling of the crystal structure into this CryoEM average suggests that a single turn of the NS2 helix is contributed by 10 monomers (Fig. 7C). The full-length NS2 oligomer showed a helical overall structure, and the N-terminal domain of NS2 contributes to the formation of the helical configuration. Two-dimensional classes with clear “ring-like” feature could be further assigned to helical NS2 oligomers on their front view (Fig. 7B and D) since their outer diameters are the same as those of “helical particles” (Fig. 7A). Interestingly, clear density could be observed inside the “ring,” which corresponds to the center of the NS2 helical shape (Fig. 7B). Considering the C-terminal end of the N-terminal domain points toward the inside of the helical particle (K160; purple sphere in Fig. 7C), we propose that the C-terminal domains of NS2 are located inside of the helical structure formed by the N-terminal domains of individual subunits of the helical oligomers of NS2.

### Disruption of putative Ca^2+^ binding residues in NS2 affects virus replication.

**FIG 8:**
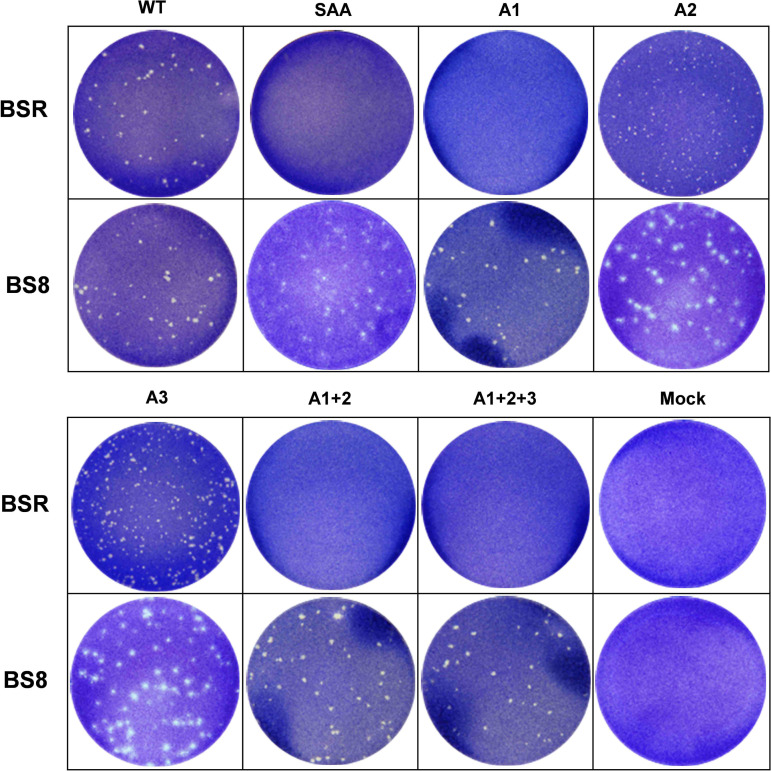
Disruption of putative Ca^2+^ binding motifs in NS2 affects plaque formation. BSR and BS8 cell monolayers were transfected with wt and each mutant S8 (Ca^2+^ binding site) together with the 9 RNA segments. SAA mutant was used as a negative control, and mock was without any transfection. Images show plaques in transfected BSR and BS8 cells.

The above data demonstrated that NS2 possesses a specific Ca^2+^ binding site. Such specific calcium binding activity is likely to influence virus fitness in infected cells. To address this issue, we introduced site directed substitution mutations in the BTV genome and studied the effect using reverse genetics (RG) as described previously (27). A set of alanine substitution mutations on NS2 were designed (Table 1). Three of these mutations in the encoding S8 segment were the same NS2 sites, A1 (DDDE_250–253_AAAA), A2 (ED_256–257_AA), or A3 (DDE_260–262_AAA). In addition, two multisite mutations in S8, A1 + A2 (DDDE_250–253_AAAA + ED_256–257_AA) and A1 + A2 + A3 (DDDE_250–253_AAAA + ED_256–257_AA + DDE_260–262_AAA) were created (Table 1) to assess if the other two sites (A2 and A3) have influence on Ca^2+^ binding residues aa 250 to 253 during virus replication. For a negative control, we used an available NS2 phosphorylation mutant SAA, in which phosphorylated serine sites (S249 and S259) were previously substituted by alanine residues (SAA) that perturbed virus replication (2, 11). When BSR cells were transfected with each mutant S8 together with 9 remaining RNA segments for virus recovery by reverse genetics, only A2 and A3 mutant viruses were recovered successfully but not A1 or the others that included the A1 mutation (A1 + A2 and A1 + A2 + A3) (Fig. 8). Subsequently, each RNA cocktail was then used to transfect BS8 cells, which stably express wtNS2 protein (segment 8) to validate the RG experiment and viability of mutant viruses. In parallel, BSR cells were also transfected similarly for comparison. Cells were fixed 48 h posttransfection. The mutant virus A2 and A3 formed plaques both in BSR and BS8 cells with similar phenotypes of wt virus and titers (PFU/ml values of ∼7 log_10_), suggesting no apparent change due to these mutations (Fig. 8). In contrast, the mutant viruses A1, A1 + A2, and A1 + A2 + A3 and the negative control SAA mutant virus showed typical plaque-forming phenotype only in the NS2 complementary BS8 cells. These data highlighted the critical role of Asp and Glu residues at aa 250 to 253 (site A1) and further validated the RG experiment of the mutant S8 that failed to recover in normal BSR cells (Fig. 8).

**FIG 9:**
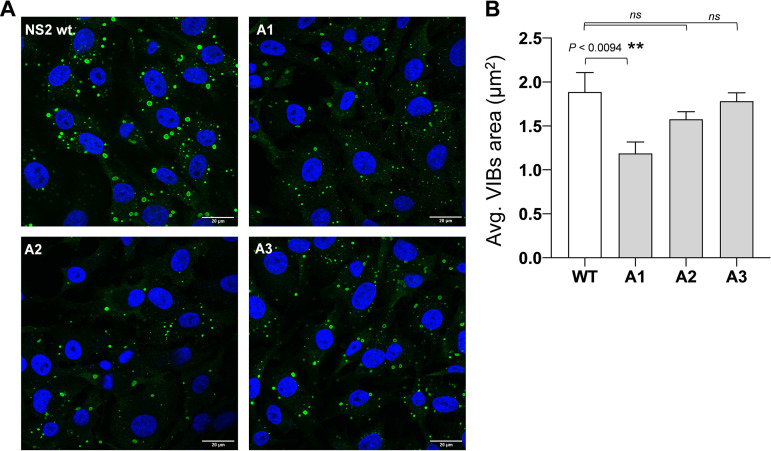
Viruses mutated in calcium binding motif showed smaller VIBs. (A) Intracellular localization of wt or mutant NS2 24 h after infection. Immunofluorescence analysis showed smaller VIBs in cells infected with the mutant A1 compared to those infected with the wt virus. NS2, green (Alexa 488); nuclei, blue (Hoechst staining). Scale bars, 20 μm. (B) Quantification of the average size of VIBs calculated as area (in μm^2^) from the microscopy data (*n* = 3). A star (*) denotes a significant difference from control (*P < *0.05). ns, Not significant.

To investigate further the failure of virus recovery with the mutation at the A1 site, we assessed whether Asp and Glu (aa 250 to 253) residues identified for Ca^2+^ sensing were critical for the NS2-triggered VIB formation, the sites of virus assembly. BSR cells were, therefore, infected with either wt virus or one of the three mutant viruses A1 (DDDE_250–253_AAAA), A2 (ED_256–257_AA), or A3 (DDE_260–262_AAA) recovered from the NS2 complementary BS8 cells, and VIB morphology in the infected BSR cells was visualized by confocal microscopy. Changes in the average size (area in μm^2^) of VIBs in cells infected with wt and mutant viruses were quantified. Cells infected with A1 mutant virus showed smaller VIBs compared to the VIBs in the wt virus-infected cells; however, mutants A2 and A3 showed no significant change in average size of VIBs (Fig. 9). The A1 mutant virus after infection in cell showed ∼1.6-fold smaller VIBs than wt virus-infected cells (Fig. 9B). Taken together, these data suggest that Ca^2+^ binding Asp and Glu residues in aa 250 to 253 have a role in VIB formation and virus replication, consistent with the *in vitro* Ca^2+^ binding and kinase assay data (Fig. 2 and 6).

### NS2 phosphorylation decreases following disruption of putative Ca^2+^ binding motifs in NS2.

**FIG 10:**
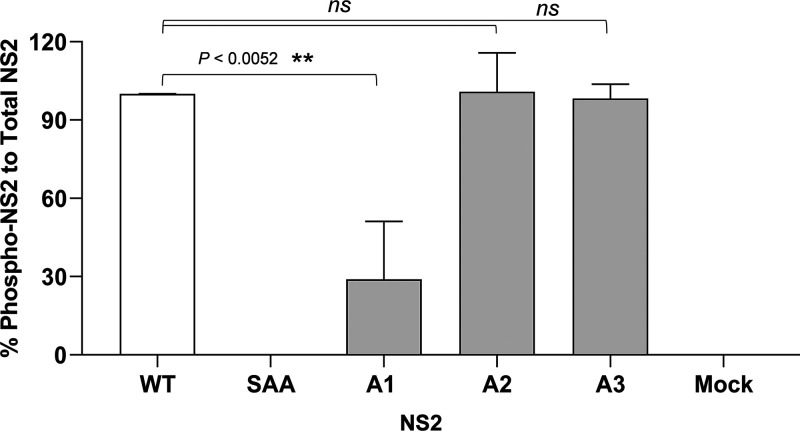
NS2 phosphorylation decreases following disruption of putative Ca^2+^ binding motifs in NS2. Quantification of NS2 phosphoproteins to total protein staining data from replicate experiments plotted in bar diagram. Error bars represent the SD values from three independent experiments. A star (*) denotes a significant difference from control (*P < *0.05) (*n* = 3). ns, Not significant.

To obtain direct evidence on whether the defective Ca^2+^ binding motif resulting in diminished VIBs was due to poor phosphorylation of NS2, BSR cells were infected with wt and the mutant viruses in addition to NS2 phosphorylation-negative mutant SAA following optimized protocol for BTV (11). BSR cells were infected with the viruses at a multiplicity of infection (MOI) of 1 and NS2 purified by immunoprecipitation (11). Pulldowns were confirmed by Western blotting and the gel stained with Pro-Q Diamond phosphoprotein gel stain followed by densitometry to determine the relative phosphorylation states. NS2 phosphorylation for mutants A2 and A3 was not significantly different from that of the wt; however, NS2 phosphorylation in mutant A1 was significantly reduced (∼70%). The SAA mutant virus showed no phosphorylation in BSR cells as previously reported (Fig. 10) (11). The decrease in phosphorylation in mutant A1 is consistent with the data obtained from *in vitro* phosphorylation experiment and poor Ca^2+^ binding observed from CD (Fig. 5 and 6). These data indicate that poor Ca^2+^ binding, due to disruption of Asp and Glu amino acid residues in the Ca^2+^ binding motif (aa 250 to 253), specifically interferes with NS2 phosphorylation in cells infected with Ca^2+^ mutant virus.

## DISCUSSION

Several viruses are known to synthesize Ca^2+^ binding proteins containing EF-hand-like motifs (28). Our *in silico* methods predicted the presence of a unique EF-hand-like calcium binding motif in NS2, resembling more closely a Ca^2+^ bowl-like structure with clusters of Asp and Glu residues. The absence of a high-resolution structure of full-length NS2 limits our understanding of the detailed structural features of the Ca^2+^ bowl. Therefore, we have taken a genetic approach to validate the predicted Ca^2+^ binding activity of this region. We tested alanine substitution of these residues by generating recombinant mutant NS2 proteins, A1, A2, and A3, targeting three sites of the putative Ca^2+^ binding region. Our biochemical and biophysical experiments with these mutant proteins demonstrated that NS2 is indeed a Ca^2+^ binding protein, and the binding of Ca^2+^ to the negatively charged residues in the A1 site (aa 250 to 253) is highly specific. Our data also suggest that the unusual Ca^2+^ bowl-like motif of NS2 acts as a Ca^2+^ sensor. In addition, in the presence of a specific chelating agent, such as EGTA, the CD spectrum of NS2-Ca^2+^ was partially reversed. Further, like several other CaBP, NS2 also demonstrated a contrasting change in the percentage of helix in NS2 (helix-to-coil) upon Ca^2+^ binding in far-UV CD spectroscopic measurements. The Ca^2+^ concentrations used in the far-UV CD experiments were to a 1:1 stoichiometry (approximately) with NS2, which is a decamer in purified form. More importantly, when we introduced the same mutations into the NS2-encoding gene of a replicating viral genome, the A1 mutation failed to recover infectious virus and plaque formation, reflecting the importance of the Ca^2+^ sensing activity of NS2 for the production of infectious virus.

To investigate the mechanism behind failure of virus recovery, we tested the effect of calcium binding mutants on the phosphorylation state of NS2 protein both *in vitro* (recombinant NS2 protein) and *in vivo* (NS2 expressed by mutant virus particles in cells). In our *in vitro* kinase assay, the level of phosphorylation was increased in the presence of Ca^2+^, indicating that Ca^2+^-mediated changes in secondary structural elements in NS2 might have led to better access to the phosphorylation sites to the enzyme. The data from the kinase assay was consistent with the CD data on Ca^2+^-mediated changes in secondary structural elements (helix) in NS2. These Ca^2+^-induced changes in NS2 are significant, although they do not appear to destabilize its oligomeric state as revealed by CryoEM analysis and size exclusion chromatography. This suggests that it is the oligomeric NS2 that senses the Ca^2+^ ions, possibly through coordination between the protomers, rather than individual monomeric subunits independently sensing Ca^2+^ ions and forming a decamer. Interestingly, a ring-like shape was inferred for full-length NS2 based on negative-stain electron microscopy (29). In this study, CryoEM analysis of full-length NS2 shows that the subunits are arranged in helical configuration (10 subunits/pitch). *In vivo*, similar effects on phosphorylation state of NS2 in cells infected with mutant virus suggest a crucial role of NS2 in host’s calcium homeostasis, which is possibly linked to the wider role of NS2 in VIB assembly/disassembly.

Several viral proteins are reported to alter cellular calcium homeostasis to assist viral life cycle by modulating mainly membrane-associated Ca^2+^ pumps. For instance, HBx of HBV modulate Ca^2+^ pumps at the plasma membrane (30), viral proteins such as Vpr (HIV-1) modulate Ca^2+^ pumps associated with mitochondrial membranes (31), and NSP4 of rotavirus (*Reoviridae*) do the same to Ca^2+^ pumps at the endoplasmic reticulum (32). It is noteworthy that, although NS2 is largely cytosolic not localizing to any membrane, it demonstrates significant potential to manipulate the host’s Ca^2+^ signaling through characteristic calcium binding motif (EF-hand-like) during the assembly of VIBs, the viral assembly factories.

Specifically, different members of the *Reoviridae* family also form similar VIB-like structures (referred to as viral factories or viroplasms in rotavirus/reovirus), although each have their own unique features (33, 34). One of the important regulatory factors critical for VIB/viroplasm assembly is phosphorylation of viral proteins, such as NSP2 of rotavirus and NS2 of BTV, which controls self-oligomerization of VIB-forming viral proteins (2, 33). Thus, our study provides further insights on the mechanism behind the regulation of this phosphorylation of VIB-forming protein NS2 in BTV. We found that Ca^2+^ binding preferentially affects phosphorylation level of the NS2, suggesting the broader relevance of NS2 in the context of modulating Ca^2+^-related signaling. During VIB assembly in the infected cells, phosphorylated NS2 is produced abundantly, and its level can be correlated with the abundance of CaBP calmodulin. These NS2 molecules play multiple roles, such as recruiting different RNA segments, self-oligomerization forming large inclusion bodies for replication, and viral genome packaging. The complex mechanism of assembly and disassembly of VIBs must be reversible and precisely regulated such that it is in sync with several other cellular processes, which is necessary to avoid any aggregate formation within the host cells during the assembly/disassembly.

Ca^2+^ signaling controls diverse cellular processes. Understanding the details of NS2-Ca^2+^ interaction greatly expands our knowledge of VIB assembly and disassembly in the context of calcium homeostasis of the cell (35–41). Although the details of the complex mechanism of VIB assembly are not well understood, the importance of phosphorylation of NS2 in VIB assembly was clearly demonstrated in our earlier study where mutations of serine residues S249 and S259 (phosphorylation site) aborted VIB assembly (2). In light of our current data, we propose a model of NS2-mediated VIB assembly in which the Ca^2+^-sensing activity of NS2 is linked to its phosphorylation status (2, 11), which in turn controls the VIB assembly required for virus replication and genome packaging.

## MATERIALS AND METHODS

### Construction of expression plasmids, protein purification, and gel filtration.

Based on the SMART motif search results (12), the plasmids for NS2 mutants were generated by the QuikChange mutagenesis method (Agilent Technologies) from the expression plasmids pET 15b-NS2 for bacterial expression and pCAG NS2 for mammalian cell expression (27, 42). Plasmids used for reverse genetics were pCAGGS BTV1 protein expression plasmids (pCAG-VP1, pCAG-VP3, pCAG-VP4, pCAG-VP6, pCAG-NS2) and T7 plasmids for BTV transcripts as previously described (27). Site-directed mutagenesis of BTV1 NS2 was performed in both T7 plasmid for the segment 8 (encoding NS2) template, pET15b-NS2, and pCAG-NS2 template using the following mutagenic primers (5′–3′): DDD_250-252_AAA (AGGTGAAGACTCTGAGTGCCGCTGCTGAACAAGGTGAGGATGC), ED_256-257_AA (CGATGATGAACAAGGTGCGGCTGCGAGTGACGATGAAC), and DDE_260-262_AAA (CAAGGTGAGGATGCGAGTGCCGCTGCACACCCAAAAACTCATA). Obtained mutants were sequenced using internal NS2 primers in order to confirm the presence of the desired mutation(s). The wild-type NS2 and mutant proteins were expressed in the E. coli bacteria strain BL21(DE3) pLysS. The culture was grown at 37°C until optical density at 600 nm (OD_600_) reached 0.5 to 0.6 and induced with 0.5 mM isopropyl-β-d-thiogalactopyranoside (IPTG). The bacterial cultures were grown at 37°C for the next 4 h postinduction. Cells were then lysed for the protein purification. Ni-nitrilotriacetic acid (Ni-NTA) affinity purification was used to purify the wild-type and mutant proteins eluting in the presence of 250 mM imidazole (elution buffer, 20 mM Tris-HCl, 150 mM NaCl, pH 7.4, 250 mM imidazole). In order to remove traces of nucleic acid from the purified protein, samples were treated with Benzonase nuclease or micrococcal nuclease where necessary. For gel filtration, a Superdex 200 10/300 GL (GE/Cytiva) column was used in a running buffer of 20 mM HEPES, 100 mM NaCl, pH 7.4, at a flow rate of 0.2 ml/min.

### Circular dichroism.

The circular dichroism (CD) spectra of wild-type apo NS2 (treated with Chelex 100 resin; Bio-Rad) were recorded in CD buffer (20 mM HEPES, 100 mM NaCl, pH 7.4) at room temperature (20°C). All buffers were pretreated with Chelex 100 resin to remove divalent metal ions contaminants. For calcium binding studies, the apo NS2 sample was titrated with Ca^2+^ salts in increasing concentration. Far-UV CD spectrum data were collected from 260 to 195 nm with a 0.5-mm rectangular cell path length at 20°C on the Applied Photophysics Chirascan and Chirascan Plus spectrometers (Leatherhead, UK) attached to a Peltier unit (Quantum Northwest TC 125). The UV and CD spectra were smoothed (window factor of 4; Savitzky-Golay method) and analyzed using Origin v6 and APL Pro-Data Viewer v4.2.15. For comparative metal ion titration experiments, same preparation of the wtNS2 protein sample was aliquoted in two halves just before titration on CD instrument. Equal amounts of Ca^2+^ and Mg^2+^ were added to each tube, respectively, in the increasing concentrations to obtain same concentrations for two metal ions. The Ca^2+^ or Mg^2+^ concentrations were taken from the 5 μM to 10 mM range. For *K_d_* measurement, CD_222 nm_ was plotted (*y* axis) for each Ca^2+^ concentration (*x* axis), and the *K_d_* value was derived using GraphPad Prism choosing inbuilt one-site total function for nonlinear regression (curve fit). Likewise, similar concentrations of mutant proteins A1, A2, and A3 were used for titration, and a similar Ca^2+^ concentration range (5 μM to 10 mM) was studied.

### *In vitro* kinase assay with [γ-^32^P]ATP.

For the kinase assay, substrate protein NS2 (6×His tag) and kinase enzyme CK2 (GST tag) were expressed separately in E. coli cells and purified using nickel and glutathione Sepharose, respectively. The kinase reaction was conducted in a 50-μl reaction mixture in 1× reaction buffer (20 mM Tris, 100 mM NaCl, pH 7.4, 1 mM dithiothreitol [DTT]). The reaction tubes were prepared by adding two different concentrations of Mg^2+^ or Ca^2+^ while keeping the substrate (0.7 mg/ml) and enzyme (0.4 mg/ml) concentrations unchanged. For other tubes, different mutants of NS2 were added with Ca^2+^. The reaction was started by adding 10 μCi of [γ-^32^P]ATP (Pekin Elmer; 250 μCi) and 0.5 mM ATP in each tube at an interval of 15 s. Additional 1× buffer was added to the tubes to adjust the dilution factor, making each tube contain up to 50 μl of reaction volume. The reaction tubes were then incubated for 30 min at 37°C. After incubation, the reaction tube was boiled in SDS sample buffer and loaded on SDS-PAGE to run at 90 V for 3 h. The SDS-PAGE gel was then dried, exposed to film, and observed under imager.

### CryoEM sample preparation, image collection, and data processing.

For CryoEM, 2.5 μl of NS2 in 20 mM Tris, 150 mM NaCl, pH 7.4, and supplemented with 0.1 mM CaCl_2_, was applied to thin continuous carbon film on lacey grid (Ted Pella) and blotted using a Vitrobot Mark IV (Thermo Fisher Scientific) with the standard Vitrobot filter paper at 4°C. The blotting time was set to 6 s, blotting force was set to 2, and drain time was set to 1 s. The grid was flash-frozen in liquid ethane and stored in liquid nitrogen. A total of 2,712 micrographs were collected on a Titan Krios 300 kV electron microscope (Thermo Fisher Scientific) equipped with a Gatan imaging filter (GIF) Quantum LS and a Gatan K2 Summit direct electron detector operated in super-resolution mode at magnification of 130K (calibrated pixel size of 0.535 Å on the sample level). The GIF slit width was set to 20 eV. The dose rate on the camera was set to ∼6.5 electrons/pixel/s. The total exposure time of each movie was 8 s, which fractionated into 40 frames of images with 0.2-s exposure time for each frame. Dose-fractionated frames were 2× binned (pixel size, 1.07 Å) and aligned for beam-induced drift correction using UCSF MotionCor2 (43). The defocus values of the micrographs were determined by CTFFIND4 (44) to be in the range of −1.0 μm and −4.0 μm. From a total of 2,712 micrographs, 463,691 particles were boxed out in 400 by 400 square pixels and 2× binned to 200 by 200 square pixels (pixel size of 2.14 Å) to speed up further data processing with Relion 3.0 (45). After one round of 2D classification, 159,361 particles (34.4% of all particles) were selected and subjected to the second round of 2D classification. Represented 2D classes are selected and used for the measurement of pitch and diameter of spiral NS2 oligomers.

### Cells, viruses, and reverse genetics.

BSR cells (BHK-21 subclone) or BS8 (BSR cells stably expressing NS2/segment of BTV) were cultured in Dulbecco’s modified Eagle’s medium (DMEM) supplemented with 5% (vol/vol) fetal bovine serum (FBS) at 35°C in 5% CO_2_. Media for BS8 was also supplemented with puromycin. Each mutant and wild-type virus were recovered (either from BSR or BS8 cells) by reverse genetics as previously described (27). Each recovered virus was plaque-purified, amplified, and titrated using plaque assay. For reverse genetics, synthetic single-stranded RNAs were prepared by runoff *in vitro* transcription from T7 PCR products using T7 RNA polymerase. Transcripts were prepared with anti-reverse cap analogue (ARCA) using mMESSAGE mMACHINE T7 Ultra kit (Ambion) as previously described (27). Reverse genetics was performed as previously described (27). Briefly, at day 1, 70 to 80% confluent BSR monolayers were transfected with pCAG-VP1, pCAG-VP3, pCAG-VP4, pCAG-VP6, and wild-type or mutated pCAG-NS2 (120 ng each) using EndoFectin (GeneCopoeia) according to the manufacturer’s instructions and incubated at 35°C in 5% CO_2_ overnight. At day 2, the cells were transfected with BTV1 exact copy RNA transcripts (S8 wild type or mutated) using EndoFectin (GeneCopoeia) overlaid with 1% agarose and incubated 3 days at 35°C in 5% CO_2_. Visible plaques were picked up and resuspended in 1% FBS containing medium, and/or cells were subsequently fixed with 10% formaldehyde and stained with crystal violet. Each recovered virus was plaque-purified, amplified, and harvested 3 days postinfection. Viruses were titrated using plaque assay.

### Immunofluorescence and VIB analysis from microscopy data.

BSR cells were grown on coverslips and infected at a multiplicity of infection (MOI) of 1 with NS2 wild-type or mutant-recovered viruses. Twenty-four hours postinfection, cells were fixed with 4% paraformaldehyde (Sigma-Aldrich) solution, permeabilized with 0.5% Triton X-100 (Sigma), blocked with 1% bovine serum albumin (BSA) (Sigma), and subsequently stained using a guinea pig anti-NS2 primary antibody (lab made) and an anti-guinea pig Alexa 488 coupled secondary antibody (Thermo Fisher Scientific). Nuclei were stained using Hoechst 33342 (Thermo Fisher Scientific). Images were acquired using an ×100 oil objective and a Zeiss Axiovert LSM 880 confocal microscope supplied with the Zen software. For each infection condition (wild-type virus versus A1, A2, or A3 mutant viruses) (Table 1), five fields were randomly selected and z-stacks (14 to 19 slices) were acquired (*x*, 1,912; *y*, 1,912; 12-bit). Each field contained in average of 17 infected cells, and the experiment was repeated three times independently. Maximum intensity projection of each z-stack was performed using Zen software, and obtained images were further processed using ImageJ software (v1.52a; https://imagej.nih.gov/ij/). Only particles (i.e., VIBs) with a size >0.5 μm^2^ were selected for particle analysis. The experiment was performed three times, and in total 1,600 VIBs were used for the wild-type virus, 764 VIBs for the DDD_250-252_AAA mutant, 1,534 VIBs for the ED_256-257_AA mutant, and 1,597 VIBs for the DDE_260-2_AAA mutant virus, respectively.

### Immunoprecipitation and phosphoprotein staining.

NS2 was purified from BTV1-infected BSR cells (MOI = 1) after 18 h. Cells were washed with phosphate-buffered saline (PBS) before lysis. Cells were lysed in lysis buffer (50 mM Tris-HCl [pH 7.5], 125 mM NaCl, 5% Glycerol, 0.2% NP-40, 1.5 mM MgCl_2_, 25 mM NaF, 1 mM Na3VO4, 1 mM beta-glycerophosphate, and 10 mM sodium pyrophosphate and protease inhibitor) for 30 min on ice. Lysates were centrifuged at 800 g for 15 min. Supernatants were recovered and added to protein A Sepharose beads conjugated to guinea-pig anti-NS2 and were incubated on ice overnight. Samples were centrifuged at 2,000 × *g* for 2 min. The supernatant was removed and the protein A Sepharose beads washed with lysis buffer. Samples were centrifuged at 2,000 × *g* for 2 min. This wash process was repeated 4 times. SDS loading buffer was then added to the protein A Sepharose beads before being boiled. SDS-PAGE gels were stained with Pro-Q Diamond phosphoprotein gel stain (Thermo Fisher). For stain, the respective fluorescence was detected and quantified.
